# Temporal Variability and Predictors of Urinary Bisphenol A Concentrations in Men and Women

**DOI:** 10.1289/ehp.10605

**Published:** 2007-11-06

**Authors:** Shruthi Mahalingaiah, John D. Meeker, Kimberly R. Pearson, Antonia M. Calafat, Xiaoyun Ye, John Petrozza, Russ Hauser

**Affiliations:** 1 Department of Obstetrics and Gynecology, Brigham and Women’s Hospital, Boston, Massachusetts, USA; 2 Department of Environmental Health Sciences, University of Michigan, Ann Arbor, Michigan, USA; 3 Department of Biostatistics, Harvard School of Public Health, Boston, Massachusetts, USA; 4 Centers for Disease Control and Prevention, Atlanta, Georgia, USA; 5 The Fertility Center, Vincent Memorial Obstetrics and Gynecology Service, Massachusetts General Hospital, Harvard Medical School, Boston, Massachusetts, USA; 6 Department of Environmental Health, Harvard School of Public Health, Boston, Massachusetts, USA

**Keywords:** bisphenol A, endocrine disruptors, environment, human, pregnancy

## Abstract

**Background:**

Bisphenol A (BPA) is used to manufacture polymeric materials, such as polycarbonate plastics, and is found in a variety of consumer products. Recent data show widespread BPA exposure among the U.S. population.

**Objective:**

Our goal in the present study was to determine the temporal variability and predictors of BPA exposure.

**Methods:**

We measured urinary concentrations of BPA among male and female patients from the Massachusetts General Hospital Fertility Center.

**Results:**

Between 2004 and 2006, 217 urine samples were collected from 82 subjects: 45 women (145 samples) and 37 men (72 samples). Of these, 24 women and men were partners and contributed 42 pairs of samples collected on the same day. Ten women became pregnant during the follow-up period. Among the 217 urine samples, the median BPA concentration was 1.20 μg/L, ranging from below the limit of detection (0.4 μg/L) to 42.6 μg/L. Age, body mass index, and sex were not significant predictors of urinary BPA concentrations. BPA urinary concentrations among pregnant women were 26% higher (–26%, +115%) than those among the same women when not pregnant (*p* > 0.05). The urinary BPA concentrations of the female and male partner on the same day were correlated (*r* = 0.36; *p* = 0.02). The sensitivity of classifying a subject in the highest tertile using a single urine sample was 0.64.

**Conclusion:**

We found a nonsignificant increase in urinary BPA concentrations in women while pregnant compared with nonpregnant samples from the same women. Samples collected from partners on the same day were correlated, suggesting shared sources of exposure. Finally, a single urine sample showed moderate sensitivity for predicting a subject’s tertile categorization.

Bisphenol A (BPA) is used to manufacture polymeric materials used for a variety of consumer products. These polymers include epoxy resins that are used to line food cans (Kang et al. 2003), polyester-styrene (Factor 1996), and polycarbonate plastics used for baby bottles and other containers (Brede et al. 2003). These polymeric resins and plastics may also be used in some dental sealants (Sasaki et al. 2005) and fillings (Joskow et al. 2006), adhesives, protective coatings, flame retardants (Samuelsen et al. 2001), and water-storage tanks and supply pipes (Bae et al. 2002). BPA is polymerized but degrades into its monomeric form over time, which can be accelerated by heat exposure (Robeson 1985). The monomeric form can leach from its source into adjacent materials, such as into water or food products. Several studies have demonstrated detectable BPA levels in packaged foods that were contained in wrapping or cans coated with BPA (Lopez-Cervantes and Paseiro-Losada 2003). Most human exposure is believed to be via ingestion (Kang et al. 2006).

Recent data have shown widespread exposure to BPA among the U.S. population. In 2,517 participants ≥ 6 years of age in the 2003–2004 National Health and Nutrition Examination Survey (NHANES III), > 92% of urine samples had detectable concentrations of BPA (Calafat et al. 2008). Other researchers have also measured detectable concentrations of BPA in a variety of human body fluids and some tissues. Of potential concern to reproductive and developmental health end points is the presence of BPA in follicular fluid and amniotic fluid (Ikezuki et al. 2002), umbilical cord blood (Schonfelder et al. 2002), and breast milk (Sun et al. 2004). In studies published as early as 1936 (Dodds and Lawson 1936), BPA has been shown to have estrogenic properties. These findings have been confirmed in several subsequent studies (Hong et al. 2006; Singleton et al. 2006). Human data on the potential health effects of BPA exposure are limited.

Adult humans metabolize BPA via hepatic glucuronidation and sulfation. The biologic half-life of BPA is approximately 6 hr, with nearly complete urinary excretion in 24 hr (Volkel et al. 2002). Therefore, urinary BPA concentrations primarily reflect exposures that occurred within the last few days preceding the collection of the urine specimen. However, because health end points of interest are likely associated with windows of exposure consisting of time intervals longer than a few days, information about the temporal variability of urinary concentrations of BPA is needed to optimize the design of exposure assessment in epidemiologic studies. Currently, published data on the temporal variability of urinary BPA concentrations are limited. Most studies include only a single urine sample that may or may not reflect an individual’s long-term exposure level. Furthermore, characteristics that may predict concentrations of BPA, such as sex, age, and body mass index (BMI), have not been explored in a large sample of adult subjects.

Temporal variability in exposure to BPA can result from changes in exposure sources, such as diet and product use, and from changes in BPA metabolism. Therefore, an individual’s BPA exposure depends on a variety of factors, and it is likely that concentrations of BPA would vary considerably over short periods, such as days. Although urinary BPA concentrations accurately measure a person’s exposure at a single point in time, determining exposure over time intervals of weeks or months requires multiple measurements. Therefore, we designed the present study to examine the temporal variability in BPA concentrations. We also explored the ability of a single urine sample to predict an individual’s longer-term exposure, over weeks or months. Finally, we also investigated the association of urinary BPA concentrations with age, BMI, sex, and pregnancy status. In regard to epidemiologic studies, this information can be used for designing exposure assessment strategies and for adjusting for measurement error in BPA exposure.

## Methods

### Subjects

Study subjects were male and female partners seeking infertility evaluation and treatment at the Massachusetts General Hospital Fertility Center. They were recruited between November 2004 and April 2006. Partners underwent ovulation induction with timed intercourse or timed intrauterine insemination and assisted reproductive technologies, which included *in vitro* fertilization and intra-cytoplasmic sperm injection. Fertility center couples that conceived naturally were also enrolled. Subjects were followed from recruitment throughout their treatment cycles until either a live birth or the discontinuation of treatment. Biochemical pregnancy was determined with a positive HCG (human chorionic gonadotropin) level (> 6.0 IU/L). Clinical intrauterine pregnancy was confirmed by the presence of a fetal heartbeat detected by trans-vaginal ultrasound scan.

The study was approved by the Human Studies Institutional Review Boards of the Massachusetts General Hospital, Harvard School of Public Health, the Centers for Disease Control and Prevention (CDC), and the University of Michigan. Subjects signed an informed consent after the study procedures were explained and all questions were answered.

Men 18–55 years of age and women 18–45 years of age were eligible. Men who had undergone a vasectomy were ineligible. Most study patients cited the lack of time as the primary reason for not participating. A research nurse administered a questionnaire to collect data on date of birth, race/ethnicity, medical history, smoking history, and lifestyle factors.

### Urine sample collection

Both men and women provided a spot urine sample at the time of recruitment and at subsequent visits during treatment cycles, as well as at post-treatment clinical appointments. Women also collected three urine samples during pregnancy, one during each trimester. Urine was collected in a nonsterile clean polypropylene container. After measuring specific gravity (SG), the urine was divided in aliquots and frozen at –80°C. Samples were shipped on dry ice overnight to the CDC.

### Urinary BPA measurements

We measured the total urinary concentration of BPA (free plus conjugated species) using online solid-phase extraction (SPE) coupled to isotope dilution–high-performance liquid chromatography (HPLC)-tandem mass spectrometry (MS/MS) on a system constructed from several HPLC Agilent 1100 modules (Agilent Technologies, Wilmington, DE) coupled to a triple quadropole API 4000 mass spectrometer (Applied Biosystems, Foster City, CA) (Ye et al. 2005). First, 100 μL of urine was treated with β-glucuronidase/sulfatase (*Helix pomatia,* H1; Sigma Chemical Co., St. Louis, MO) to hydrolyze the BPA-conjugated species. BPA was then retained and concentrated on a C18 reversed-phase size-exclusion SPE column (Merck KGaA, Germany), separated from other urine matrix components using a pair of monolithic HPLC columns (Merck KGaA), and detected by negative ion-atmospheric pressure chemical ionization-MS/MS. The limit of detection (LOD) for BPA in a 0.1-mL urine sample was 0.36 μg/L. Low-concentration (~ 4 μg/L) and high-concentration (~ 20 μg/L) quality control materials, prepared with pooled human urine, were analyzed with standard, reagent blank, and unknown samples (Ye et al. 2005). BPA concentrations < LOD were assigned a value equal to one-half the LOD (Hornung and Reed 1990) prior to adjustment by SG.

The first 25 urine samples collected were analyzed for the concentration of the free species of BPA using a method similar to the one described above, but without β-glucuronidase/sulfatase treatment. In agreement with published reports (Volkel et al. 2002), the percentage of free BPA was essentially zero. Therefore, for the remainder of the urine samples, we measured only the total BPA concentration.

Several methods are used to adjust for urine volume (Boeniger et al. 1993; Teass et al. 1993). However, because organic compounds, such as phenols, that are glucuronidated in the liver are eliminated by active tubular secretion (Boeniger et al. 1993), creatinine adjustment may not be appropriate (Teass et al. 1993). Additionally, creatinine concentrations may be confounded by muscularity, physical activity, urine flow, time of day, diet, and disease states (Boeniger et al. 1993; Teass et al. 1993). For these reasons, we used SG rather than creatinine. Urinary BPA concentrations were normalized for dilution using the formula *P_c_* = *P* × [(1.024 – 1)/(*SG* – 1)], where *P_c_* is the SG-corrected BPA concentration (in micrograms per liter), *P* is the observed BPA concentration (in micrograms per liter), and *SG* is the specific gravity of the urine sample (Boeniger et al. 1993; Teass et al. 1993). SG was measured using a handheld refractometer (National Instrument Company Inc., Baltimore, MD), which was calibrated with deionized water before each measurement.

### Statistical analysis

Descriptive statistics and distributions of urinary BPA concentrations were tabulated and compared among demographic categories. We constructed graphs to visually and qualitatively compare BPA concentrations within and between subjects over time. Using SAS software (version 9.1; SAS Institute Inc., Cary, NC), mixed effects models were fit to determine the association of urinary BPA concentrations (log_10_) with age, BMI, sex, and pregnancy status. Each model included the predictor of interest and SG as fixed effects. To account for possible correlation of measurements, random effects were included initially for subject, couple, and within-couple specimen collection date. The variance component for couple was estimated to be zero and was dropped from the models, but the random effects for subject and within-couple collection date were retained. Full covariate data were available for all specimens, so information from all samples was used in the analyses.

To explore the nature of within-couple correlation, we regressed the geometric mean of SG-adjusted BPA measurements from the female partner on that from the male partner. Another analysis compared BPA measurements (SG-adjusted and log_10_-transformed) of the female to those of the male when the specimens were collected on the same date. In this model, a random effect was included to account for a possible correlation due to repeated measurements from the same couple.

We calculated the sensitivity, specificity, and positive predictive value of a single urine sample for predicting high BPA tertile by comparing predicted and observed classifications for agreement (Hauser et al. 2004; Meeker et al. 2005). Positive predictive value is the probability that a person is actually in the exposure group of interest, given that they were classified in that group based on the single urinary BPA value that was available. BPA tertiles were determined for the SG-adjusted BPA concentrations among all 82 subjects. For those with more than one sample, we used the subject’s geometric mean value in the tertile classification. A contingency table was then constructed to display the level of agreement between each subject’s “true” tertile classification as determined by the geometric mean value of their repeated samples and their tertile classification predicted by each of their single repeat samples. Only subjects with three or more urine samples (31 subjects with a total of 149 urine samples) were included in the contingency tables. After combining contingency tables for all subjects with three or more samples, we calculated sensitivity, specificity, and positive predictive value for the ability of a given single urine sample to classify a subject in the highest BPA tertile.

We then calculated the sensitivity, specificity, and positive predictive value of two urine samples from an individual to classify subjects in the highest BPA tertile. This analysis was limited to subjects with at least six repeat urine samples (*n* = 8 subjects who contributed a total of 67 samples). Geometric means of all possible combinations of sample pairings from each individual were used in the analysis as the predicted value. The geometric mean value from all possible within-subject combinations was then compared with the geometric mean value of their repeated samples, used to determine their “true” BPA tertile classification. As above, contingency tables were then constructed. The goal of the validity analysis was to simulate and compare the ability of exposure assessments that involve one or two urine samples to predict a subject’s “true” longer-term exposure.

In both of these analyses, the single or two urine samples used to predict a subject’s tertile classification are not independent predictors of their overall geometric mean value because they are also used to calculate the subject’s geometric mean. For instance, when we calculated the geometric mean for subjects with three or more urine samples, the single urine sample evaluated for its predictive ability was included in the geometric mean calculation for that subject. Because of this, we limited these analyses to subjects with three (or six) or more urine samples to minimize the structural dependence between the predicted value based on the single sample and the “true” observed values based on the geometric mean of that subject’s full complement of samples.

## Results

Urinary BPA concentrations were measured in 217 samples collected from 82 subjects. On average, each subject contributed 2.6 urine samples, ranging from 1 (*n* = 34 subjects) to 13 samples (*n* = 1 subject). Twenty-nine (13%) urine samples had BPA concentrations < LOD. Forty-five women contributed 145 urine samples and 37 men contributed 72 specimens. Among subjects, 24 women (93 specimens) and 24 men (53 specimens) were partners. Of the urine samples from these 24 couples, 42 pairs of specimens were time-matched (collected on the same day). Eleven couples had two or more time-matched urine samples, and 10 couples had a single time-matched urine sample. Three couples had no time-matched urine samples.

The study subjects ranged in age from 28 to 54 years, with a mean ± SD of 35.5 ± 4.4 years (Table 1). Seventy subjects (85%) were Caucasian. Only five subjects (three men and two women) were current smokers. BMI (in kilograms per square meter) ranged from 16.5 to 44.7, with a mean ± SD of 26.8 ± 5.8. Ten of the women became pregnant during the follow-up period, as confirmed by the presence of a fetal heart beat on ultrasound. One pregnancy loss occurred.

Among the 217 urine samples, the median BPA concentration was 1.20 μg/L (25th percentile, 0.60; 75th percentile, 2.70), with a range from < LOD of 0.4 μg/L to 42.6 μg/L. However, because the number of urine samples collected from subjects varied widely, subjects contributed differentially to the median and percentiles of the urinary BPA concentrations. For instance, a subject with four urine samples contributed four times as many data points as a subject with one sample. In addition, multiple urine samples from a given subjects are not independent. To account for both of these factors, we calculated the percentiles of the urinary BPA distribution based on the geometric mean of the urinary BPA concentrations for each subject with two or more urine samples and the single urine sample concentration for subjects with one sample (Table 2). After accounting for these factors, the median was 1.30 μg/L (25th percentile, 0.72; 75th percentile, 2.09). The median was slightly lower among women (1.08 μg/L) than among men (1.40 μg/L). Urinary concentrations unadjusted for SG (Table 2) allow for comparisons with other study populations, because few studies have used SG to adjust for urinary dilution.

Because spot urine samples were collected at different times throughout the day, we explored whether time of day was associated with urinary SG-adjusted BPA concentrations (Table 3). Urine samples collected before 0900 hours (time of collection ranged from 0530 to 0859 hours) had a median of 2.4 μg/L compared with medians of 1.92 μg/L for samples collected from 0900 to 1159 hours, whereas samples collected between noon and 1559 hours had a higher median of 3.24 μg/L. Median BPA then decreased to 1.62 μg/L among samples collected after 1600 hours (range 1600 to 1830 hours). In mixed regression analysis, there was a statistically significant difference in geometric mean SG-adjusted BPA concentration by time of day when urine samples were collected (*p* = 0.01 for comparison of samples collected between noon and 1559 hours with those collected after 1600 hours).

Although age, BMI, sex, and pregnancy status were not statistically significant predictors of urinary BPA concentrations, we found weak associations after reverse transformation of the regression coefficients. Controlling for SG, BPA concentrations increased by 2% [95% confidence interval (CI), –2 to 6%) for each year age increased. To explore associations of BMI with urinary BPA concentrations, BMI was categorized as underweight (< 20), normal (20–24.9), overweight (25–29.9), or obese (≥ 30). Compared with normal BMI, underweight BMI was associated with a 49% increase (95% CI, –25 to 197%) in BPA concentration, overweight BMI with an 8% increase (95% CI, –27 to 61%), and obese BMI with a 3% decrease (95% CI, –38 to 51%). BPA concentrations for women were 9% lower (95% CI, –35 to 28%) than those for men, and concentrations for urine samples collected from women while pregnant were 26% higher (95% CI, –26 to 115%) than urine samples collected from nonpregnant women. Restricting to the 10 women who became pregnant during follow-up, samples collected while women were pregnant had concentrations 33% higher (95% CI, –33 to 165%) than those collected prepregnancy (*n* = 49 samples).

To investigate within-couple correlation of urinary BPA concentrations, we first noted that the covariance parameters in the mixed-effects models were estimated to be approximately 0.04 for subject, 0.04 for within-couple collection date, and 0.11 for residual variance. Because the variance component for couples had been estimated to be zero, these numbers suggest that BPA concentrations between partners will correlate substantially only when the measurements are taken on the same day. As a second approach, we used the geometric mean of SG-adjusted BPA concentrations as an average exposure measurement for each individual; using ordinary linear regression, we compared the female’s geometric mean with that of her male partner (*n* = 24 couples). We observed no significant association between the means (Spearman correlation = 0.04, *p* = 0.84); similar results were found using arithmetic means or arithmetic means on the log_10_ scale (*p* = 0.92 and 0.84).

**Figure 1:**
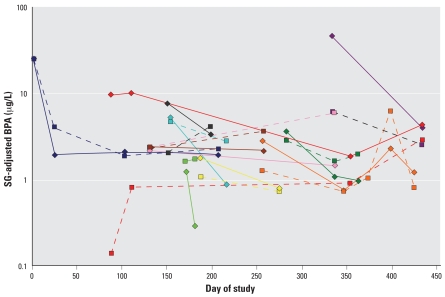
Within-couple variability shown by urinary SG-adjusted BPA concentrations among female (diamonds and solid lines) and male (squares and broken lines) partners with at least two urine samples collected on the same day (*n* = 11 couples).

However, when female BPA concentration (SG-adjusted and log_10_-transformed) on a particular day was compared with that of her male partner on the same day (*n* = 42 pairs of day-matched, couple-matched specimens), a moderately strong association was found (Figure 1). Using a linear mixed model accounting for correlation from repeated observations on the same couple by inclusion of random effects, each one unit increase in male log_10_ SG-adjusted BPA urine concentration was estimated to correspond to a 0.428 increase (95% CI, 0.110 to 0.747) in the female partner’s urine BPA concentration from the same day (Spearman correlation = 0.36; *p* = 0.02).

Among subjects with at least three urine samples, the calendar time between collection of the first and last urine samples ranged from 14 to 482 days, with a mean (median) of 172 (150) days. The time between collection of first and last urine samples ranged from 150 to 482 days for subjects with at least six samples, with a mean (median) of 304 (329) days.

Sensitivity and specificity analyses to assess the ability of one urine sample to predict a subject’s exposure tertile are shown in Table 4. The proportion of subjects who “truly” were in the highest exposure tertile (top 33%), identified as those with a single urine sample collected anytime during the subject’s participation in the study (i.e., sensitivity) was 0.64. The proportion of subjects who “truly” were in the lowest exposure tertiles (e.g., first and second tertiles) and classified as such (i.e., specificity) was 0.76. The proportion of single urine samples that classified an individual in the highest tertile who was “truly” classified in the highest group (i.e., positive predictive value) was 0.63. We also calculated these measures for the ability of two urine samples to predict an individual’s geometric mean (Table 4). When two BPA samples were used to classify each subject, we found increases in sensitivity (0.67), specificity (0.84), and positive predictive value (0.85).

## Discussion

Apart from a small study in Japan on five subjects (four men and one woman) (Arakawa et al. 2004), we report some of the first data on long-term temporal variability of urinary BPA concentrations among a large sample of both men and women. We found temporal within-subject variability in urinary SG-adjusted BPA concentrations. As suggested by earlier small studies, we also found that men had slightly higher BPA concentrations than women. Japanese researchers reported associations of male sex and androgen levels with higher BPA concentrations (Takeuchi and Tsutsumi 2002; Takeuchi et al. 2004). However, Kim et al. (2003) found that BPA urine concentrations in males and females were similar. Further exploration of a potential sex- or androgen-related difference in urinary BPA concentrations is needed to resolve the inconsistencies across limited studies.

To the best of our knowledge, the present study is the first to compare urinary BPA concentrations in women before and during pregnancy. Although only 10 women contributed data, there was a nonsignificant 33% increase in urinary concentrations of BPA during pregnancy compared with prepregnancy. During pregnancy, there are numerous physiologic changes that may affect BPA distribution, metabolism, and/or clearance. For instance, the glomerular filtration rate is increased during pregnancy, creatinine clearance increases, and in some pregnant women, urine output increases due to increased metabolism of antidiuretic hormone. To explore whether changes in urinary concentrations of BPA are partially due to changes in urine SG during pregnancy, we regressed SG on pregnancy status, clustering by subject. The estimated SG for pregnant women was 0.0029 higher than for nonpregnant women (95% CI, –0.0014 to 0.0071; *p* = 0.19). Therefore, changes in SG during pregnancy would contribute to a slightly lower SG-adjusted BPA concentration, rather than an increase, as we observed during pregnancy.

Other potential explanations for differences in urinary BPA concentrations during pregnancy include *a*) changes in exposure, such as an increased use of products or in the consumption of foods that contain BPA; *b*) changes in volume of distribution and/or altered sequestration of BPA into different body fluid compartments, such as amniotic fluid; and *c*) changes in BPA metabolism and/or excretion. Application of physiologically based pharmacokinetic approaches is necessary to better understand changes in BPA dynamics during pregnancy, but it is beyond the scope of the present report.

Because exposure to BPA is thought to be primarily via ingestion, we hypothesized that urinary BPA concentrations will be more similar among individuals that are partners than those who are not partners. We found evidence of a moderately strong relationship between urinary BPA concentrations in samples collected on the same day among male and female partners. This suggests that BPA exposure occurs through a common household lifestyle factor, most likely diet. This finding warrants further investigation because sources of BPA among the general population are unclear.

Interestingly, there appeared to be a trend in urinary BPA concentrations by the time of day when the sample was collected. Urinary BPA concentrations were highest in the samples collected between 1200 and 1600 hours, compared with morning or late afternoon/ evening samples. This may reflect dietary BPA exposure during the midday meal, because BPA has a short half-life (Volkel et al. 2002). We did not collect data on the time of day meals or foods were consumed throughout the day, so we are unable to confirm this directly.

We also conducted sensitivity and specificity analyses to determine the ability of one urine sample or a pair of urine samples to predict long-term urinary BPA concentrations over several months. Despite the presence of temporal variability in urinary BPA concentrations, our sensitivity and specificity analyses suggested that a single urine sample correctly classified subjects into the highest BPA exposure tertile based on the geometric mean of repeat samples for that subject. However, there were several limitations to our approach. First, because there was no gold standard to use as the “true” urinary BPA concentration, we relied on using the geometric mean of repeat urine sample concentrations. How well these geometric means represent an individual’s true exposure is unclear. Furthermore, there is structural dependency between the “true” urinary BPA concentrations and the single or pair of urine samples that will over-inflate the sensitivity and specificity. Second, the timing of the collection of the repeat urine samples was not balanced. For instance, the duration between the collection of consecutive repeat urine samples ranged from 3 days to a year or more, but all samples were treated equally without respect to the time between collection. Thus, although we found that two urine samples improved the ability to predict BPA exposure tertiles, the preferred temporal spacing of the two samples is currently unclear. However, based on previous work on other nonpersistent chemicals, such as phthalates, at least 1 month between samples is preferable when the exposure duration of interest is over several months (Hauser et al. 2004).

Despite a much longer span of time between the collection of the first and last urine samples among subjects in the two-sample sensitivity analysis, the predictive ability was increased with the collection of a second sample. For the subset included in the two-sample sensitivity analysis (subjects with at least six samples), the average time interval between samples was 304 days compared with 175 days among the group of subjects included in the one-sample sensitivity analysis (subjects with at least three samples). Because samples from the two-sample sensitivity analysis represent a much wider exposure period, we would not have been surprised by a lack of improvement in the two-sample analysis compared to the one-sample analysis. However, our finding of increased predictive ability in the two-sample analysis may suggest that, because BPA exposure is ubiquitous and likely occurs daily, urinary concentrations exist in a relatively pseudosteady state over the course of months or years (National Research Council 2006).

The urinary BPA concentrations found in the present study were within the range reported from studies in several countries, including Japan, Korea, and the United States. In a nonrepresentative subset of NHANES III that included men and women between 20 and 59 years of age, the geometric mean urinary BPA concentration was 1.33 μg/L (Calafat et al. 2005). Among girls 6–8 years of age, Wolff et al. (2007) reported a geometric mean of 2.0 μg/L. Yang et al. (2003) reported a geometric mean urinary BPA concentration of 9.54 μg/L among 73 Koreans (34 men and 39 women) In another Korean study on 15 men and 15 women, Kim et al. (2003) reported a mean (SE) urinary BPA concentration of 2.82 μg/L (0.73) and 2.76 μg/L (0.54), respectively, with no difference by sex. Among 48 women in Japan, Ouchi and Watanabe (2002) reported a median urinary BPA concentration of 1.2 μg/L, with a range of 0.2–19.1 μg/L. Comparisons across studies are difficult because they may be confounded by differences in timing and methods of urine collection, containers used, analytical methods to quantify BPA, different limits of detection, and methods for correction of urinary dilution. Additionally, country-specific regulations on the use of BPA and occupational exposure to BPA may affect urinary concentrations. Countries with bans on the use of BPA products for packaging foods may have populations with lower urinary and serum BPA concentrations. However, we are not aware of data showing population differences in exposure before and after bans.

## Conclusion

Despite within-person variability in urinary BPA concentrations, a single sample is predictive of long-term exposure (over weeks to months) and provides good sensitivity to classify individuals into tertiles in epidemiologic studies. The addition of a second urine sample improved this classification ability. Although we did not find strong relationships of urinary BPA concentrations with age, sex, BMI, and pregnancy status, these associations were suggestive and are worthy of further follow-up. Discovery of whether urinary concentrations increase during pregnancy, reflecting either increased exposure or alterations in metabolism during pregnancy, is especially important. The correlation between urinary BPA concentrations among male and female partners suggests that BPA exposure is shared through diet or common residential source(s). Finally, replication of this study in other populations, including the general population, should be conducted to determine whether predictors vary across populations. In addition, further work needs to be done to determine the utility of urine samples to define an individual’s exposure level during pregnancy.

## Figures and Tables

**Table 1:** Subject demographics.

Characteristic	All subjects (*n* = 82)	Males (*n* = 37)	Females (*n* = 45)
Age [years (mean ± SD)]	35.5 ± 4.4	35.7 ± 5.3	35.2 ± 3.6
BMI (mean ± SD)	26.8 ± 5.8	28.2 ± 5.2	25.7 ± 6.1
Race [*n* (%)]			
Caucasian	70 (85)	30 (81)	40 (89)
African-American	0 (0)	—	—
Asian	7 (9)	4 (11)	3 (7)
Other	5 (6)	3 (8)	2 (4)
Smoking history [*n* (%)]			
Never	56 (68)	27 (73)	29 (64)
Former	21 (26)	7 (19)	14 (31)
Current	5 (6)	3 (8)	2 (4)

**Table 2:** Distribution of urinary BPA concentrations (μg/L).

			Percentiles						
	No.	GM	10th	25th	50th	75th	90th	95th	Max
Initial urine sample only									
All subjects	82	1.31	0.18	0.60	1.30	2.70	5.00	5.80	42.6
Male	37	1.62	0.60	1.00	1.40	3.10	5.10	6.50	18.7
Female	45	1.09	0.18	0.50	0.90	2.20	4.80	5.80	42.6
All urine samples^a^									
All subjects	82		0.33	0.72	1.30	2.09	4.16	4.80	12.8
Male	37		0.50	0.92	1.40	3.08	4.50	5.00	6.5
Female	45		0.25	0.50	1.08	1.80	4.16	4.80	12.8

Abbreviations: GM, geometric mean; Max, maximum. Levels < LOD were recorded as 0.18 (1/2 LOD). Urinary concentrations were not adjusted for SG to allow for comparison with other studies in the literature.

a The percentile distributions were based on the GM of the urinary BPA concentrations for each subject with two or more urine samples (48 subjects) and the single urine sample concentration for subjects with one sample (34 subjects).

**Table 3:** Median (25th and 75th percentiles) and geometric mean SG-adjusted BPA concentrations (μg/L) grouped by time of urine sample collection.

Time	No.	Median (25th and 75th percentile)	Geometric mean
0530–0859 hours	49	2.40 (1.20, 4.32)	2.37
0900–1159 hours	106	1.92 (1.07, 3.49)	2.01
1200–1559 hours	52	3.24 (1.96, 5.32)	3.42
1600–1830 hours	10	1.62 (0.80, 2.40)	1.42

**Table 4:** Sensitivity, specificity, and positive predictive value of one or two urine samples to predict the highest BPA tertile.^a^

	Sensitivity	Specificity	Positive predictive value
One sample^b^	0.64	0.76	0.63
Two samples^c^	0.67	0.84	0.85

a BPA tertile cutoff values (μg/L): < 1.61; ≥ 1.61 to < 3.11; ≥ 3.11.

b Analysis included 149 samples from 31 subjects with at least three repeat urinary BPA measures.

c Analysis included 67 samples from 8 subjects with at least six repeat urinary BPA measures.
